# Cathepsin S Alters the Expression of Pro-Inflammatory Cytokines and MMP-9, Partially through Protease—Activated Receptor-2, in Human Corneal Epithelial Cells

**DOI:** 10.3390/ijms19113530

**Published:** 2018-11-09

**Authors:** Wannita Klinngam, Runzhong Fu, Srikanth R. Janga, Maria C. Edman, Sarah F. Hamm-Alvarez

**Affiliations:** 1 Department of Pharmacology and Pharmaceutical Sciences, School of Pharmacy, University of Southern California, Los Angeles, CA 90007, USA; klinngam@usc.edu (W.K.); runzhonf@usc.edu (R.F.); 2 Department of Ophthalmology, Roski Eye Institute, Keck School of Medicine, University of Southern California, Los Angeles, CA 90007, USA; janga@usc.edu (S.R.J.); edman@usc.edu (M.C.E.)

**Keywords:** Sjögren’s syndrome, Cathepsin S, pro-inflammatory cytokines, proteases, protease-activated receptor-2, matrix metalloproteinase 9, inflammatory dry eye, human corneal epithelial cells, ocular surface inflammation

## Abstract

Cathepsin S (CTSS) activity is increased in tears of Sjögren’s syndrome (SS) patients. This elevated CTSS may contribute to ocular surface inflammation. Human corneal epithelial cells (HCE-T cells) were treated with recombinant human CTSS at activity comparable to that in SS patient tears for 2, 4, 8, and 24 h. Acute CTSS significantly increased HCE-T cell gene and protein expression of interleukin 6 (IL-6), interleukin 8 (IL-8), tumor necrosis factor-α (TNF-α), and interleukin-1β (IL-1β) from 2 to 4 h, while matrix metalloproteinase 9 (MMP-9), CTSS, and protease-activated receptor-2 (PAR-2) were increased by chronic CTSS (24 h). To investigate whether the increased pro-inflammatory cytokines and proteases were induced by CTSS activation of PAR-2, HCE-T cells were transfected with PAR-2 siRNA, reducing cellular PAR-2 by 45%. Cells with reduced PAR-2 expression showed significantly reduced release of IL-6, TNF-α, IL-1β, and MMP-9 into culture medium in response to acute CTSS, while IL-6, TNF-α, and MMP-9 were reduced in culture medium, and IL-6 and MMP-9 in cell lysates, after chronic CTSS. Moreover, cells with reduced PAR-2 expression showed reduced ability of chronic CTSS to induce gene expression of pro-inflammatory cytokines and proteases. CTSS activation of PAR-2 may represent a potential therapeutic target for amelioration of ocular surface inflammation in SS patients.

## 1. Introduction

Sjögren’s syndrome (SS) is a systemic autoimmune disease with hallmark symptoms associated with lymphocytic infiltration and loss of function of lacrimal gland (LG) and salivary gland, resulting in dry eye and dry mouth symptoms [1]. Lymphocytic infiltration of the LG and release of pro-inflammatory cytokines can affect the ocular surface by impairing the neural network responsible for modulating tear secretion, resulting in reduced tear flow and production of inflammatory and proteolytic tears [2,3]. One of the consequences of inflammation of the ocular surface in SS is enhanced apoptosis and autophagy of the cells of the ocular surface system [4]. A recent study has shown that increased autophagy marker expression in tears and conjunctival epithelium have positive correlations with ocular surface damage [4]. The corneal epithelium is a part of the ocular surface system greatly affected by the development and progression of SS. The corneal epithelium of SS patients is poorly lubricated and its irregular surface can enhance tear film instability. SS patient corneal epithelium also exhibits evidence of impaired corneal epithelial barrier function that can be detected by corneal fluorescein staining [3]. SS patients who have suffered from dry eye for more than 10 years show higher amounts of corneal fluorescein staining, suggesting that ocular surface damage is associated with SS progression [2]. The reduction of tear volume, tear film instability, and ocular surface inflammation in SS patients are all associated with significant patient discomfort and impaired vision due to decreased corneal sensation and neural damage from inflammation [2,5]. SS patients are at a higher risk for sight-threatening ocular manifestations such as corneal ulceration, corneal perforation, cicatrizing conjunctivitis, uveitis, optic neuritis, scleritis, or retinal vasculitis [6].

Cathepsin S (CTSS), a lysosomal protease, participates in many physiological processes including protein catabolism, major histocompatibility complex class II - antigen presentation and extracellular matrix degradation [7,8]. We have previously demonstrated that CTSS is elevated specifically in the tears of SS patients relative to patients with other autoimmune diseases or SS-independent dry eye [9]. CTSS is implicated in the pathology of other inflammatory and autoimmune diseases such as atherosclerosis [10,11], rheumatoid arthritis [12], multiple sclerosis [13], bronchial asthma and chronic obstructive pulmonary disease [14]. Since CTSS is associated with inflammation and autoimmune responses in other diseases, our overall hypothesis was that elevated tear fluid CTSS activity in SS might affect corneal and ocular surface homeostasis.

More specifically, it has been established that the balance of cytokines in tear fluid and conjunctival epithelium is altered in SS patients. Significantly increased expression of IL-1α, IL-6, IL-8, TNF-α, and TGF-β1 was found in conjunctiva epithelium of SS patients when compared to healthy controls [15]. Other studies demonstrate that IL-1, IL-6, IL-8, TNF-α, interferon-γ (IFN-γ) and IL-17 are highly expressed in SS patient tears relative to non-SS patients [16,17,18,19], suggesting that elevated pro-inflammatory cytokines may be related to disease progression in SS patients. In this study, we specifically hypothesized that tear CTSS may directly increase pro-inflammatory cytokine expression in human corneal epithelial cells, an effect that may contribute to ocular surface inflammation in SS.

Matrix metalloproteinase 9 (MMP-9) is a protease responsible for remodeling extracellular matrix, and is associated with pathology in diverse inflammatory diseases including arthritis, cardiovascular diseases, pulmonary diseases, cancer, systemic lupus erythematosus, and SS [20]. MMP-9 protein expression and enzymatic activity are elevated in LG of female MRL/lpr and male Non Obese Diabetic (NOD) mice, murine models of SS [21]. MMP-9 activity is also highly increased in SS patient tears when compared to healthy controls [17], linking increased MMP-9 with ocular surface pathogenesis. Additionally, some studies have reported an association between MMP-9 and pro-inflammatory cytokines. For instance, increased IL-6, IL-8, IL-1β, and TNF-α can induce MMP-9 expression in malignant non-Hodgkin’s lymphoma, human neutrophils, and corneal epithelial cells respectively [22,23,24,25]. MMP-9 itself may elicit expression of IL-1β, TNF-α, and TGF-β, which may then perpetuate the inflammatory cycle [3,26]. In this study, we hypothesized that increased CTSS in tears might also induce MMP-9 in human corneal epithelial cells.

Protease–activated receptor-2 (PAR-2) is a G-protein-coupled receptor activated by specific proteases such as trypsin, mast cell tryptase, factor Xa and VIIa, and other serine proteases. PAR-2 is activated through cleavage of its extracellular domain, generating a tethered ligand which induces downstream intracellular signaling pathways [27]. Recent studies link activated PAR-2 to inflammatory processes associated with respiratory, gastrointestinal, metabolic, cardiovascular, and neurological diseases [28]. PAR-2 is expressed in a variety of cells such as keratinocytes [29], endothelial cells [30], fibroblasts [31], neurons [32], immune and inflammatory cells [33] and also in epithelial cells such as lung, gastrointestinal tract and corneal cells [34,35,36]. Recent studies have shown that CTSS can also cleave PAR-2 near the N-terminus at a site distinct from trypsin, exposing a novel tethered ligand and leading to the release of inflammatory mediators linked to several pathological conditions including hyperexcitability of nociceptive neurons promoting neurogenic inflammation and neuropathic pain [37], endothelial cell injury [38,39], visceral hyperalgesia during colitis [40], induction of chronic atopic dermatitis [41], and activation of liver tumor-initiating cells associated with hepatocellular carcinoma [42]. Several studies primarily conducted with serine proteases such as trypsin, mast cell tryptase, and bacterial proteases, arginine proteases, and elastase link activated PAR-2 to induction of pro-inflammatory cytokines. For example, activated PAR-2 leads to the release of IL-6 and IL-8 in oral epithelial cells, keratinocytes, sebocytes, and corneal epithelial cells [43,44,45,46], and also stimulates mRNA expression of IL-1 and TNF-α in keratinocytes, endothelial cells, and sebocytes [29,46,47,48]. PAR-2 activation by trypsin increases MMP-9 expression and enzymatic activity in airway epithelial cells and keratinocyte [29,49]. As a final component of our hypothesis, we speculated that tear CTSS might be able to induce inflammatory responses in corneal epithelial cells through activation of corneal epithelial PAR-2.

To test these hypotheses regarding the ability of tear CTSS to induce pro-inflammatory cytokines and MMP-9, and the role of PAR-2 in this process, we utilized cultured human corneal epithelial cells treated with recombinant human CTSS at activity levels approximating those found in SS patient tears, and measured changes in pro-inflammatory cytokines, MMP-9 and CTSS itself utilizing qPCR and ELISA. We have further explored the role of PAR-2 in the process utilizing siRNA to reduce its expression in vitro and to probe changes in these downstream indicators of inflammation. 

## 2. Results

### 2.1. Acute CTSS Exposure Significantly Increases IL-1β, IL-8, IL-6, and TNF-α Gene and Protein Expression in Cell Culture Medium and Cell Lysates of Human Corneal Epithelial Cells

**Figure 1:**
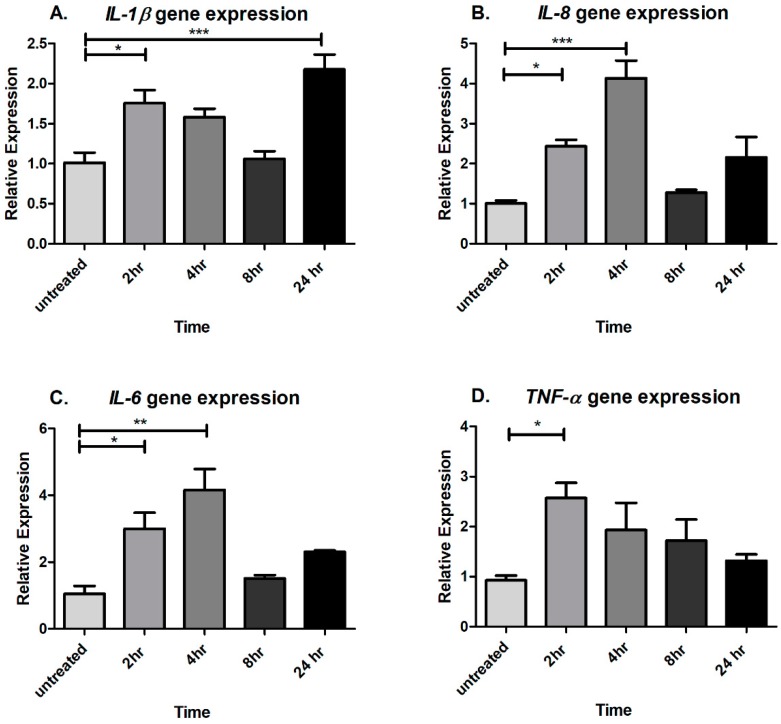
CTSS increases *IL-1β*, *IL-8*, *IL-6*, and *TNF-α* gene expression after 2- and 4-hours of treatment in a human corneal epithelial cell line (HCE-T cells) (**A**) *IL-1β* gene expression without and with CTSS treatment in HCE-T cells; (**B**) *IL-8* gene expression without and with CTSS treatment in HCE-T cells; (**C**) *IL-6* gene expression without and with CTSS treatment in HCE-T cells; (**D**) *TNF-α* gene expression without and with CTSS treatment in HCE-T cells. The amount of CTSS added corresponded to an activity level found in the 90th–95th percentile of SS patients (18,000 RFU, added to 500 µL of cell medium), as described in detail in **Methods**. Expression of genes of interest were normalized to expression of the endogenous gene, *GAPDH* (*n* = 3 samples/group, * *p* ≤ 0.05, ** *p* ≤ 0.01, *** *p* ≤ 0.001, data are represented as mean ± SEM and one-way ANOVA with Dunnett’s multiple comparison was used to compare treated to untreated cells).

To study whether CTSS affects pro-inflammatory cytokine gene expression, human corneal epithelial cells from the HCE-T cell line were treated with recombinant human CTSS at an enzymatic activity comparable to that we detect at the 90th–95th percentile (18,000 Relative Fluorescence Units or RFU per 500 µL of cell medium) in SS patient tears [9] (see **Methods** for a detailed explanation). Cells were treated with CTSS for 2, 4, 8, and 24 h. The cell viability after 24 h of CTSS treatment was 97.3 ± 5.2% (mean ± SEM) relative to untreated cells (*n* = 3). Then, gene expression of pro-inflammatory cytokines of interest was measured and compared to untreated cells. The results indicate that CTSS can enhance *IL-1β*, *IL-8*, *IL-6*, and *TNF-α* gene expression after acute exposure (2 to 4 h) (Figure 1A–D). *IL-1β* gene expression was significantly increased after 2 and after 24 h of CTSS treatment (Figure 1A). *IL-8* and *IL-6* gene expression began to increase after 2 h of treatment and showed the highest expression at 4 h of treatment (Figure 1B,C). Additionally, CTSS significantly increased *TNF-α* gene expression after 2 h of treatment **(**Figure 1D).

**Figure 2:**
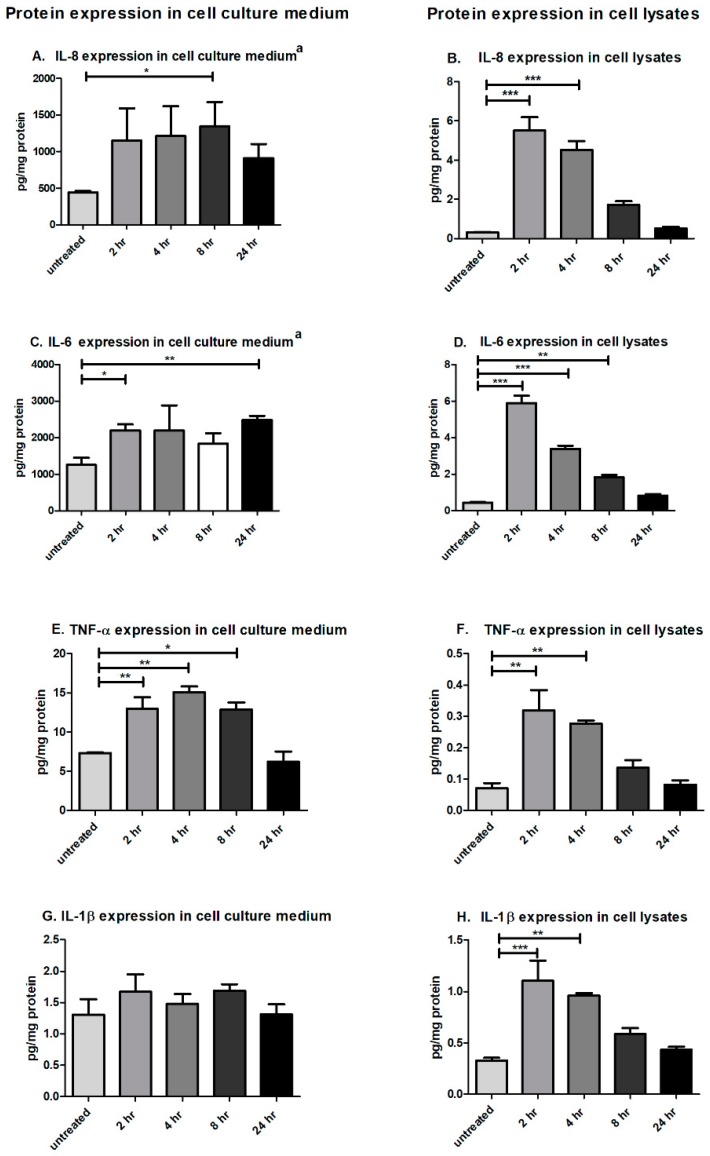
CTSS significantly increases IL-8, IL-6, and TNF-α, IL-1β protein expression in cell culture medium and cell lysates from human corneal epithelial cells (HCE-T cells) at 2, 4, and 8 h of exposure. (**A**) IL-8 protein expression in cell culture medium from HCE-T cells without and with CTSS; (**B**) IL-8 protein expression in cell lysates from HCE-T cells without and with CTSS; (**C**) IL-6 protein expression in cell culture medium from HCE-T cells without and with CTSS; (**D**) IL-6 protein expression in cell lysates from HCE-T cells without and with CTSS; (**E**) TNF-α protein expression in cell culture medium from HCE-T cells without and with CTSS; (**F**) TNF-α protein expression in cell lysates from HCE-T cells without and with CTSS; (**G**) IL-1β protein expression in cell culture medium from HCE-T cells without and with CTSS; (**H**) IL-1β protein expression in cell lysate from HCE-T cells without and with CTSS. The amount of CTSS added corresponded to an activity level found in the 90th–95th percentile of SS patients (18,000 RFU, added to 500 µL of cell medium), as described in detail in **Methods**. Expression of proteins of interest were normalized to total protein concentration of respective cell culture medium or lysates. a = individual ELISA kit (*n* = 3 samples/group, * *p* ≤ 0.05, ** *p* ≤ 0.01, *** *p* ≤ 0.001, data are represented as mean ± SEM and one-way ANOVA with Dunnett’s multiple comparison was used to compare treated to untreated cells).

To confirm whether CTSS affected protein expression comparably to gene expression, the Pro-inflammatory Panel 1 (human) Multiplex assay kit (MSD^®^, Rockville, MD, USA), which allows quantitation of up to 10 pro-inflammatory cytokines in the same sample, was used to analyze the protein expression of pro-inflammatory cytokines in cell culture medium and cell lysates in HCE cells treated with CTSS for 2, 4, 8, and 24 h, compared to untreated cells. Protein expression results largely corresponded with gene expression data, showing that CTSS increased IL-8, IL-6, and TNF-α protein expression in both cell culture medium and cell lysates at 2, 4, and 8 h of treatment (Figure 2A–F). Although no significant induction of IL-1β protein expression was noted in cell culture medium, CTSS still significantly increased IL-1β protein expression in cell lysates after 2 and 4 h of treatment (Figure 2G,H). In addition, CTSS increased *IL-1β* gene expression in cell lysates and IL-6 protein expression in cell culture medium after cells were treated with CTSS for 24 h, suggesting that there might be a later phase of cytokine responsiveness to chronic exposure to this protease.

### 2.2. Chronic CTSS Exposure Significantly Increases MMP-9 Gene Expression

**Figure 3:**
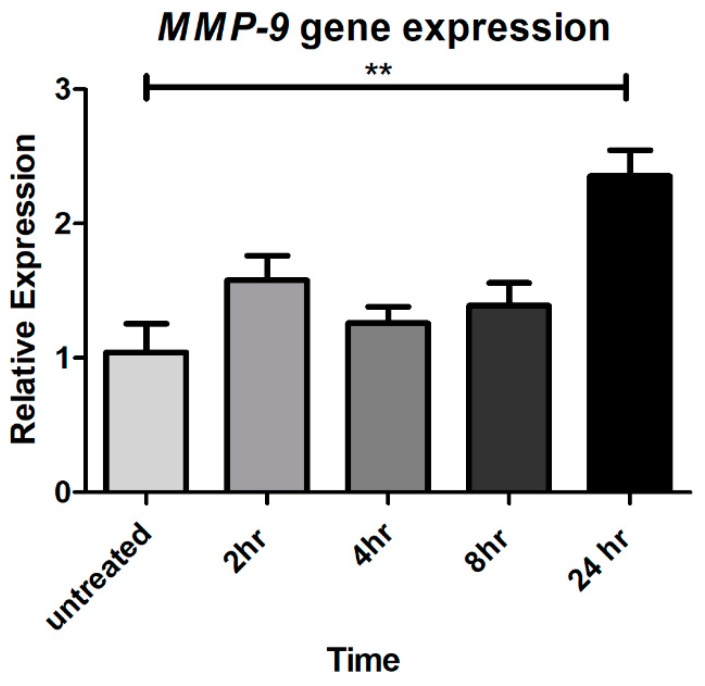
CTSS increases *MMP-9* gene expression after 24 h in human corneal epithelial cells. *MMP-9* gene expression in HCE-T cells without and with CTSS. The amount of CTSS added corresponded to an activity level found in the 90th–95th percentile of SS patients (18,000 RFU, added to 500 µL of cell medium), as described in detail in **Methods**. *MMP-9* gene expression was normalized to expression of the endogenous gene, *GAPDH* (*n* = 3 samples/group, ** *p* ≤ 0.01, data are represented as mean ± SEM and one-way ANOVA with Dunnett’s multiple comparison was used to compare treated to untreated cells).

To investigate whether CTSS can induce MMP-9 expression in corneal epithelial cells, possibly enhancing extracellular matrix degradation and ocular surface inflammation, gene expression was analyzed in HCE-T cells without and with treatment with CTSS for 2, 4, 8, and 24 h. The results show that *MMP-9* gene expression is significantly elevated after 24 h of CTSS relative to levels in untreated cells (Figure 3).

### 2.3. Chronic CTSS Exposure Significantly Increases PAR-2 Gene and Protein Expression

**Figure 4:**
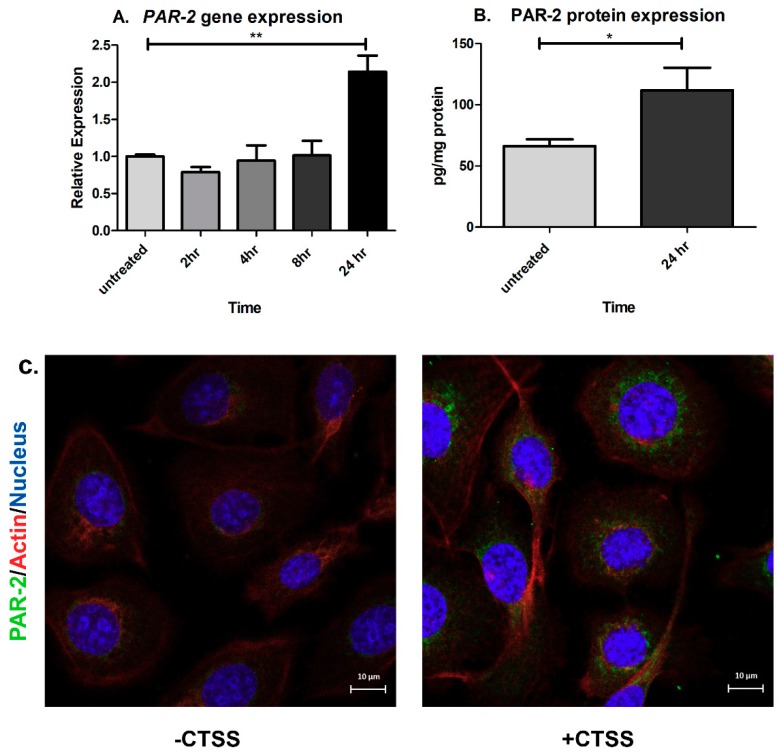
CTSS increases PAR-2 gene and protein expression after 24 h in human corneal epithelial cells. (**A**) *PAR-2* gene expression in HCE-T cells without and with CTSS. *PAR-2* gene expression was normalized to expression of the endogenous gene, *GAPDH* (*n* = 3 samples/group and one-way ANOVA with Dunnett’s multiple comparison was used to compare treated to untreated cells); (**B**) PAR-2 protein expression measured by using the human PAR-2 ELISA assay in HCE-T cell lysates without and with CTSS. PAR-2 protein expression was normalized to total protein in lysates (*n* = 4 samples/group and a two-tailed, unpaired Student’s *t*-test was used to compare treated to untreated cells); (**C**) HCE-T cells treated without and with CTSS for 24 h and fixed and processed using primary and secondary antibodies to detect PAR-2 by indirect immunofluorescence. The amount of CTSS added corresponded to an activity level found in the 90th–95th percentile of SS patients (18,000 RFU, added to 500 µL of cell medium), as described in detail in **Methods**. (* *p* ≤ 0.05, ** *p* ≤ 0.01 and data are represented as mean ± SEM).

CTSS can cleave and activate PAR-2, promoting neurogenic inflammation and pain in the skin and intestine [32,50,51,52]. To investigate first whether CTSS affects PAR-2 expression in human corneal epithelial cells, HCE T-cells were treated with recombinant CTSS for 2, 4, 8, and 24 h. Then, gene and protein expression of PAR-2 was measured and compared to untreated cells. We found that PAR-2 gene and protein expression were significantly increased after 24 h of CTSS treatment relative to untreated cells (Figure 4A–C). *PAR-2* gene expression was significantly increased by 2-fold (Figure 4A), which corresponded to a 1.7-fold increased PAR-2 protein expression measured by ELISA relative to untreated cells (Figure 4B). In addition, when PAR-2 protein expression was analyzed using indirect immunofluorescence, CTSS-treated cells showed a higher intensity of PAR-2 immunofluorescence relative to untreated cells (Figure 4C). These findings suggest that CTSS can increase PAR-2 expression at both the gene and protein levels after 24 h of exposure.

### 2.4. CTSS Exposure Significantly Increases CTSS Gene and Protein Expression at 8- and 24-h

**Figure 5:**
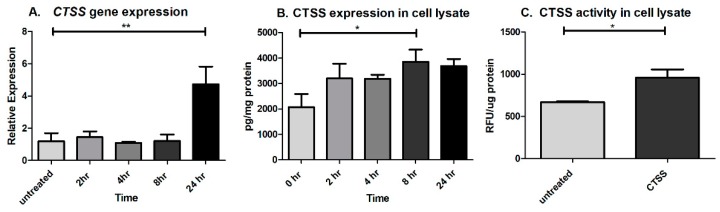
CTSS exposure in human corneal epithelial cells increases cellular CTSS gene and protein expression after 8- and 24-h (**A**) *CTSS* gene expression in HCE-T cells without and with CTSS. *CTSS* gene expression was normalized to expression of the endogenous gene, *GAPDH*. One-way ANOVA with Dunnett’s multiple comparison was used to compare treated to untreated cells; (**B**) CTSS protein expression measured by using the human CTSS human ELISA assay in HCE-T cell lysates without and with CTSS treatment at different time points. CTSS protein expression was normalized to total protein in lysates and a one-way ANOVA with Dunnett’s multiple comparison was used to compare treated to untreated cells; (**C**) Enzymatic CTSS activity after 24 h in HCE-T cells without and with CTSS. Enzymatic CTSS activity was normalized to total protein in lysates and a two-tailed, unpaired Student’s *t*-test was used to compare treated to untreated cells. (*n* = 3 samples/group, * *p* ≤ 0.05, ** *p* ≤ 0.01, data are represented as mean ± SEM). The amount of CTSS added corresponded to an activity level found in the 90th–95th percentile of SS patients (18,000 RFU, added to 500 µL of cell medium), as described in detail in **Methods**.

Recent studies have demonstrated that intracellular CTSS can also affect expression of pro-inflammatory cytokines. CTSS-overexpressing mice showed higher expression of Th2-type cytokines such as IL-4 and IL-10, and also increased levels of Th1-type cytokines such as TNF-α, IFN-γ, and IL-1β [41]. CTSS from splenic dendritic cells induced IL-6 production in response to systemic exposure to lipopolysaccharide [53]. Both extracellular and intracellular CTSS can also activate IL-36γ, a newly discovered IL-1 family member, and can induce the secretion of IL-8 in a human keratinocyte cell line [54]. Our findings of later or sustained elevations in some pro-inflammatory cytokines and MMP-9 after CTSS treatment suggested that there might be pathways that stimulate their later induction, possibly a positive feedback loop involving increased CTSS expression itself after 24 h. To test our hypothesis, CTSS gene and protein expression were analyzed after 2, 4, 8, and 24 h of CTSS treatment. The results showed that *CTSS* gene expression was increased after cells were treated with recombinant CTSS for 24 h (Figure 5A). CTSS protein expression in cell lysates measured using the human CTSS ELISA kit also revealed that the protein was increased after 8 h of CTSS treatment (Figure 5B). In addition, CTSS activity in cell lysates was significantly increased after 24 h of CTSS treatment, relative to untreated cells (Figure 5C). These data suggest that CTSS exposure induces its own gene and protein expression, in a way that may promote a positive feedback loop, which contributes further to a cycle of induction of pro-inflammatory cytokines as has been reported in other systems.

### 2.5. Acute Exposure to Heat-Inactivated CTSS Does not Induce Pro-Inflammatory Cytokines

**Figure 6:**
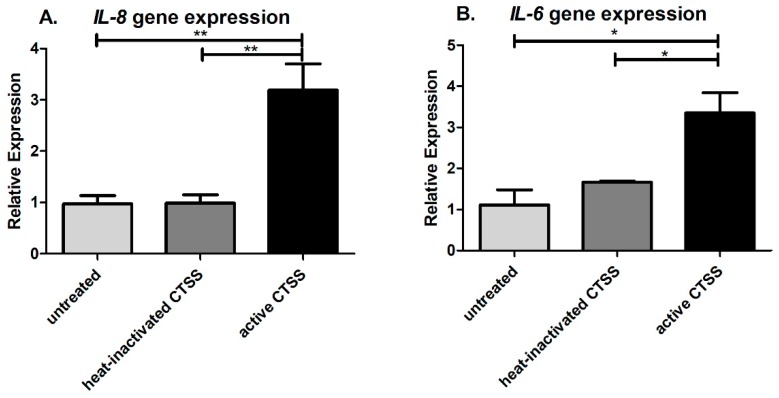
CTSS activity is required for early induction of pro-inflammatory cytokines in human corneal epithelial cells. (**A**) *IL-8* gene expression in HCE-T cells without (untreated), with heat-inactivated CTSS, and with active CTSS; (**B**) *IL-6* gene expression in HCE-T cells without (untreated), with heat-inactivated CTSS, and with active CTSS. *IL-8* and *IL-6* gene expression were normalized to expression of the endogenous gene, *GAPDH* (*n* = 3 samples/group, * *p* ≤ 0.05, ** *p* ≤ 0.01, data are represented as mean ± SEM, and one-way ANOVA with Tukey’s multiple comparison was used to compare cells within different CTSS treatments. The amount of CTSS added corresponded to an activity level found in the 90th–95th percentile of SS patients (18,000 RFU, added to 500 µL of cell medium), as described in detail in **Methods**. Heat inactivation was by heating at 90 °C for 30 min.

As shown in Figure 1, 2 to 4 h of CTSS treatment was sufficient to induce maximal pro-inflammatory cytokine gene and protein expression. To investigate whether CTSS activity was critical for this early induction of pro-inflammatory cytokine expression, heat-inactivated CTSS was used to treat HCE T-cells for 4 h, and the gene expression of *IL-8* and *IL-6* after 4 h treatment was measured and compared to that elicited in HCE -T cells treated with active CTSS under the same conditions. Recombinant human CTSS was inactivated by heating at 90 °C for 30 min, resulting in reduction of its activity from 364 RFU/µL to 1 RFU/µL. *IL-8* gene expression in cells treated with heat-inactivated CTSS was not significantly different relative to untreated cells, but was significantly reduced relative to levels in cells treated with active CTSS (Figure 6A). Similarly, *IL-6* gene expression showed no difference between untreated cells and cells exposed to heat-inactivated CTSS, but a statistically significant increase in *IL-6* gene expression in cells treated with active CTSS versus cells treated with heat-inactivated CTSS or untreated cells (Figure 6B). These findings suggest that CTSS activity is required for the early induction of pro-inflammatory cytokines. 

### 2.6. CTSS-Dependent Activation of PAR-2 Activation Is Involved in Induction of Pro-Inflammatory Cytokines and Proteases in Human Corneal Epithelial Cells

**Figure 7:**
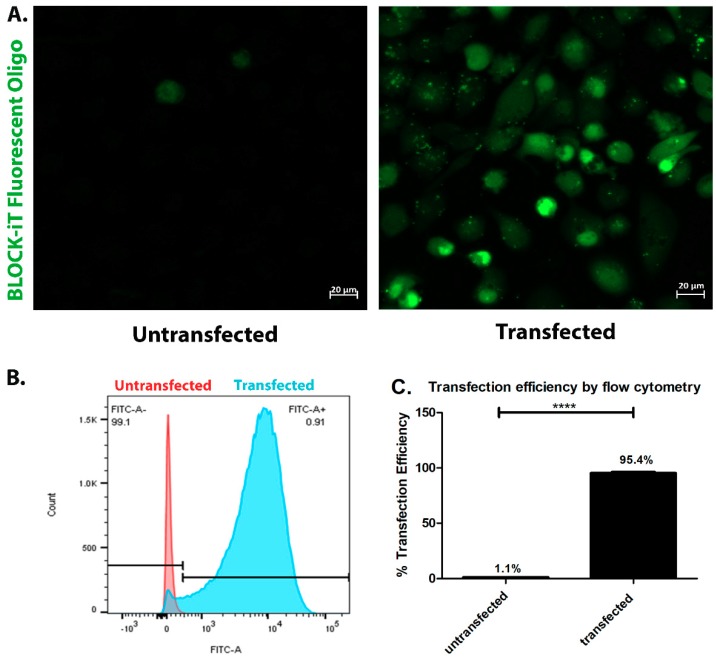
Transfection efficiency of siRNA using BLOCK-iT Fluorescent Oligo labeled with FITC in human corneal epithelial cells after 48 h of incubation. (**A**) Cellular distribution of BLOCK-iT Fluorescent Oligo labeled with FITC in transfected HCE-T cells compared to untransfected cells; (**B**) The flow cytometry histogram from transfected cells compared to untransfected cells. The black bar represents the ranged gate in which 99.1% of untransfected cells were determined as FITC negative and 0.9% of untransfected cells were determined as FITC positive; (**C**) Transfection efficiency measure by flow cytometry in transfected HCE-T cells relative to untransfected cells (*n* = 3 samples/group, **** *p* ≤ 0.0001, data represented as mean ± SEM, and a two-tailed, unpaired Student’s *t*-test was used to compare transfected to untransfected cells).

**Figure 8:**
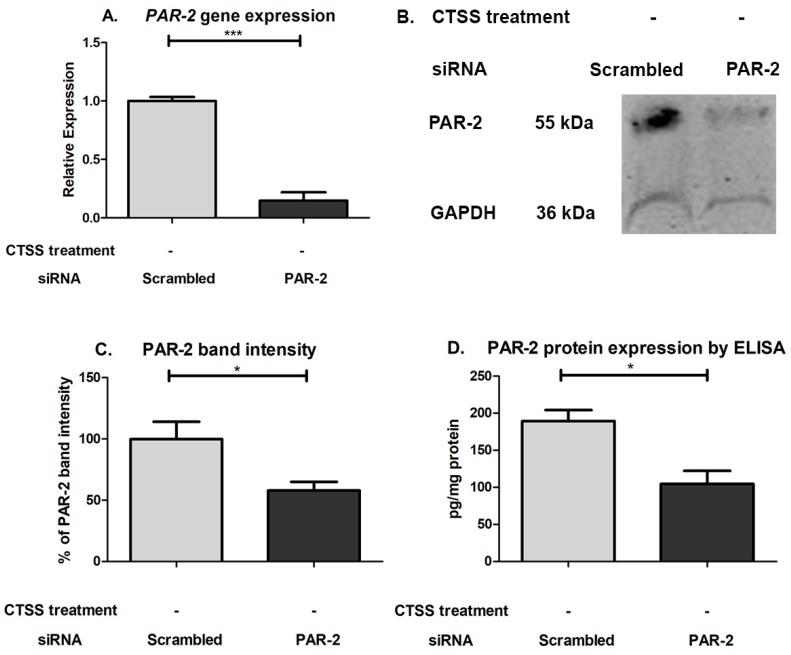
PAR-2 gene and protein expression after 48 h of PAR-2 or scrambled siRNA transfection in human corneal epithelial cells. (**A**) *PAR-2* gene expression in HCE-T cells transfected with PAR-2 or scrambled siRNA. *PAR-2* gene expression was normalized to expression of the endogenous gene, *GAPDH* (*n* = 3 samples/group; (**B**) PAR-2 bands measured by Western Blotting in lysates from human corneal epithelial cells transfected with PAR-2 or scrambled siRNA; (**C**) PAR-2 band intensity in human corneal epithelial cells transfected with PAR-2 or scrambled siRNA. The intensity signal of PAR-2 band was normalized to the band intensity of GAPDH and designated as 100% for scrambled siRNA-treated cells (*n* = 5 samples/group); (**D**) PAR-2 protein expression in HCE-T cells transfected with PAR-2 or scrambled siRNA as determined by ELISA. PAR-2 protein expression was normalized to total protein in lysates (*n* = 3 samples in PAR-2 siRNA transfected and *n* = 2 in scrambled siRNA transfected, * *p* ≤ 0.05, *** *p* ≤ 0.001 data are represented as mean ± SEM, and a two-tailed, unpaired Student’s *t*-test was used to compare PAR-2 siRNA transfected to scrambled siRNA transfected cells).

To investigate whether the activation of PAR-2 by CTSS is responsible for the induction of pro-inflammatory cytokines and proteases associated with exposure of cultured corneal epithelial cells to CTSS, human PAR-2 siRNA was used to reduce cellular PAR-2 expression before treatments with recombinant CTSS. The transfection efficiency of siRNA in HCE-T cells was determined with the BLOCK-iT Fluorescent Oligo labeled with FITC. As shown in Figure 7A–C, the transfection efficiency was 95.4%. The efficiency of PAR-2 knockdown using PAR-2 siRNA was evaluated by the analysis of PAR-2 expression by analyzing gene and protein expression. The results showed that after HCE-T cells were transfected with 25 pmol of PAR-2 siRNA for 48 h, a ~77% reduction of *PAR-2* gene expression was achieved relative to scrambled siRNA-transfected cells, which showed no reduction in gene expression (Figure 8A). The PAR-2 band migrates from 44–55 kDa. In our study, we observed a principal immunoreactive band at 55 kDa (Figure 8B). The PAR-2 band intensity in cells transfected with scrambled and PAR-2 siRNA was normalized to the band intensity of GAPDH, used as a loading control. We found that the PAR-2 band intensity in cells transfected with PAR-2 siRNA showed a 42% reduction compared to cells transfected with scrambled siRNA (Figure 8C), which corresponded with ELISA results that showed a 45% reduction in cells transfected with PAR-2 siRNA relative to cells transfected with scrambled siRNA (Figure 8D). This suggests that although the mRNA is markedly reduced, cellular PAR-2 protein may turn over more slowly within the experimental time frame of 48 h siRNA treatment.

**Figure 9:**
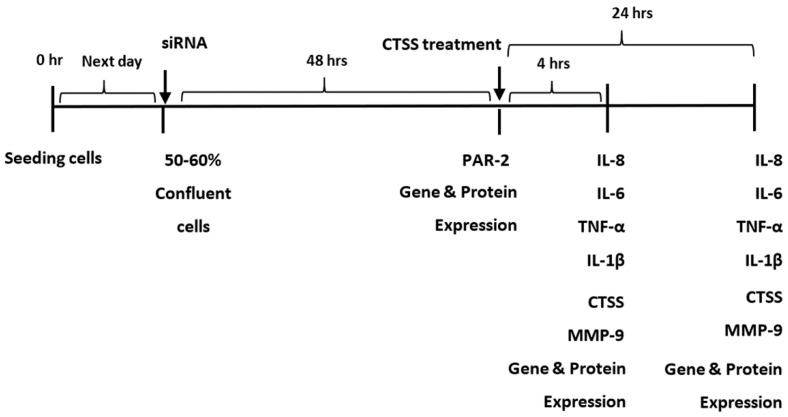
Experimental design to probe the effect of CTSS activation of PAR-2 on the increase in pro-inflammatory cytokines (IL-8, IL-6, TNF-α, and IL-1β) and proteases (CTSS and MMP-9) at 4 and 24 h of recombinant CTSS treatment.

Cells were then treated with siRNA treatment to reduce PAR-2 expression and were exposed to CTSS to 4 or 24 h, representing acute and chronic exposures (Figure 9). Gene (Table 1) and protein (Table 2) expression of pro-inflammatory cytokines of interest (IL-1β, IL-8, IL-6, TNF-α) and proteases (CTSS and MMP-9) were measured according to the experimental protocol shown in Figure 9 (with the exception of CTSS in culture medium which was not measured since recombinant CTSS treatments interfered with measurement of CTSS released from the cells). Only *IL-6* and *TNF-α* gene expression in cells transfected with PAR-2 siRNA were reduced relative to scrambled siRNA transfected cells at 4 h of CTSS treatment. However, after 4 h of CTSS treatment, PAR-2 siRNA transfected cells showed reduced secretion of IL-6, TNF-α and IL-1β into culture medium relative to scrambled siRNA treated cells, with the effect persisting in culture medium for IL-6 and TNF-α at 24 h of CTSS treatment. IL-6 was the only pro-inflammatory cytokine with cellular levels in lysates that showed a reduction at 4 h of CTSS treatment after PAR-2 knockdown. These changes all occurred at times independent of CTSS-induced changes in PAR-2 protein expression, suggesting that they are mediated under normal conditions by existing plasma membrane PAR-2 in the absence of any CTSS induction. In contrast, *IL-8* gene expression in cells transfected with PAR-2 siRNA was unaffected at 4 h of CTSS treatment, and no significant changes in its secretion to cell culture medium or content in cell lysates was seen at either time point relative to cells transfected with scrambled siRNA. Transfection of cells with PAR-2 siRNA decreased MMP-9 secretion into culture medium both at 4 and 24 h, and also decreased MMP-9 protein expression in cell lysates at 4 h of CTSS treatment. Expression of all genes of interest was decreased in cells treated with PAR-2 siRNA relative to scrambled siRNA by 24 h of exposure to CTSS. These findings suggest that PAR-2, which is known to be activated by CTSS, is important for the early CTSS-dependent induction of *IL-6* and *TNF-α* gene expression (Table 1), the increased acute CTSS-dependent secretion of IL-6, IL-1β, TNF- α and MMP-9 protein (Table 2), and possibly the later CTSS-dependent changes in gene expression of many of these factors (Table 1).

### 2.7. An Initial PAR-2 Dependent Increase in TNF-α after Acute CTSS Exposure May Drive IL-6 and IL-1β Gene Expression in Human Corneal Epithelial Cells

**Figure 10:**
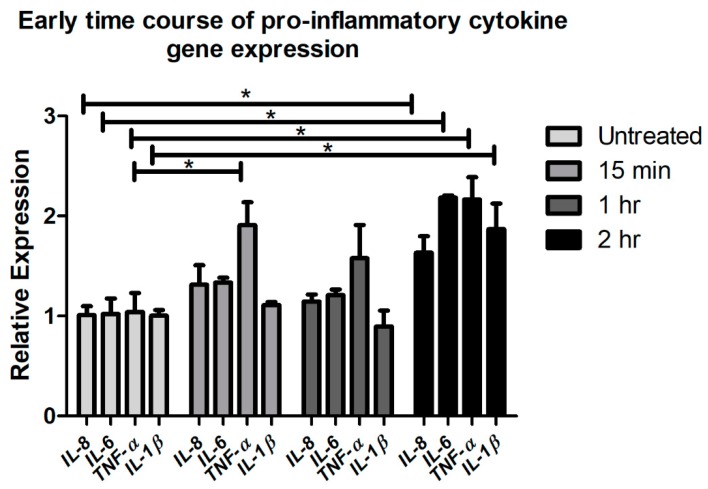
Gene expression of pro-inflammatory cytokines (*IL-8*, *IL-6*, *TNF-α*, and *IL-1β*) after 15 min, 1 h, and 2 h of CTSS treatment in human corneal epithelial cells (HCE-T cells). The amount of CTSS added corresponded to an activity level found in the 90th–95th percentile of SS patients (18,000 RFU, added to 500 µL of cell medium), as described in detail in **Methods**. Expression of genes of interest was normalized to expression of the endogenous gene, *GAPDH* (*n* = 3 samples/group, * *p* ≤ 0.05, data are represented as mean ± SEM and one-way ANOVA with Dunnett’s multiple comparison was used to compare treated to untreated cells).

Some studies have reported that TNF-α can induce IL-6 and IL-1β expression via the TNFRI receptor and p38 mitogen-activated protein kinase (MAPK), phosphoinositide 3-kinase (PI3K)/Akt and nuclear factor (NF)-κB pathways [55]. To investigate whether an early CTSS-mediated and PAR-2 dependent increase in TNF-α could theoretically drive PAR-2 independent elevations in IL-6 and IL-1β, we explored the time course of gene expression of pro-inflammatory cytokines from Figure 1 at 15 min, 1 h and 2 h of CTSS treatment relative to untreated cells. Figure 1 and Figure 2 show that changes in gene and protein expression of these cytokines occur in parallel under these conditions. Figure 10 shows that only *TNF-α* gene expression was increased after 15 min of CTSS treatment relative to untreated cells, while expression of the other pro-inflammatory cytokines was increased only after 2 h of CTSS treatment. Since increased expression of TNF-α temporally precedes that of IL-6 and IL-1β and is PAR-2 dependent, it is therefore possible that it may drive the downstream changes in IL-6 and IL-1β, which may be PAR-2 independent but TNF-α-dependent.

## 3. Discussion

Concurrent with and sometimes even preceding the lymphocytic infiltration characteristic of SS, altered levels of pro-inflammatory cytokines and other inflammatory mediators are found in LG and salivary glands of SS mouse models, and also in glands of SS patients [56,57,58]. These mediators are also found in tears and conjunctival epithelium [15], and it is thought that their release from the inflamed LG may impact the ocular surface. The activity of CTSS, a cysteine endopeptidase, is highly increased in the tears of SS patients [9,59]. Here, we show that CTSS is also able, at activity levels found in SS patient tears, to acutely stimulate pro-inflammatory gene and protein expression in a human corneal epithelial cell line. Thus, tear and ocular surface tissue cytokines may not necessarily originate in the LG in SS but may be elicited via CTSS-mediated processes in ocular surface epithelia.

**Figure 11:**
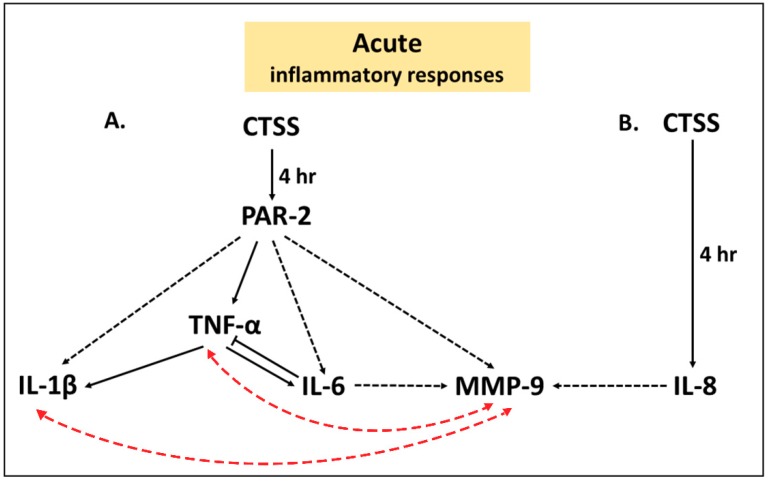
The possible cell signaling pathways induced by acute exposure to 4 h of CTSS in human corneal epithelial cells contributing to increased release of pro-inflammatory cytokines and MMP-9 that may contribute to ocular surface inflammation. (**A**) The effects of acute CTSS on increased pro-inflammatory cytokines and MMP-9 mediated by PAR-2; (**B**) Acute CTSS induces IL-8 through a PAR-2- independent pathway. A one-way black solid arrow (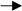) represents a proposed inducible pathway, an one-way black dashed arrow (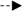) represents a possible one-way inducible pathway, a two-way red dashed arrow (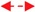) represents a possible two-way inducible pathway and a solid line with blunt end (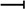) represents a negative feedback pathway.

Our findings further implicate PAR-2 as a potential mediator of acute CTSS—induced ocular surface inflammatory responses. In HCE-T cells transfected with PAR-2 siRNA but not scrambled siRNA and followed by acute exposure to 4 h of CTSS treatment, secretion of IL-6, TNF-α, IL-1β, and MMP-9 into cell culture medium were significantly reduced, corresponding with decreased *IL-6* and *TNF-α* gene expression and IL-6 and MMP-9 protein content in cell lysates. This effect occurred prior to the CTSS-induced increase in PAR-2 protein expression at 24 h, suggesting it is mediated by PAR-2 already present on or recruited to the cell’s surface. Collectively these changes suggest that CTSS activation of PAR-2 may play a possible role in priming of acute inflammatory responses (Figure 11).

Our time course studies also suggested that the early CTSS-mediated and PAR-2-dependent increase in TNF-α by 15 min might subsequently contribute through PAR-2 independent mechanisms to elevated IL-6 and IL-1β (Figure 11). In addition, the effects elicited by acute CTSS exposure on pro-inflammatory cytokines and MMP-9 may be interrelated. Previous studies have shown that both IL-1β and TNF-α can upregulate MMP-9 in corneal epithelial cells, again through the NF-κB and p38 pathways or by upregulating other mediators that regulate MMP-9 [24,25]. Furthermore, IL-6 can induce MMP-9 expression in human malignant non-Hodgkin’s lymphomas [22], and IL-8 can induce MMP-9 expression in human neutrophils through activation of the CXCR2 receptor [23]. Additionally, MMP-9 can itself stimulate IL-1β and TNF-α, possibly creating a positive feedback cycle accelerating ocular surface inflammation [3,60]. Our experiments cannot currently distinguish whether the acute phase increase in IL-6, IL-1β and MMP-9 that appears to be PAR-2-dependent are direct or influenced by changes in TNF-α as well as cross-talk between pathways as indicated (Figure 11).

While CTSS caused an acute increase in *IL-6*, *IL-8*, and *IL-1β* gene expression at 2 and 4 h, this elevation was reduced by 8 h but was then again increased after 24 h, suggestive of a cyclical spiking of inflammatory responses. The pathway creating this positive feedback might be related to the mutual induction of MMP-9 and pro-inflammatory cytokines that happens in the acute phase. Not only MMP-9 but also CTSS itself is increased by CTSS exposure, thus creating a positive feedback loop, possibly leading to more severe ocular surface inflammation. Finally, CTSS induces PAR-2 expression after 24 h of exposure in HCE-T cells, suggesting its upregulation as a chronic consequence of CTSS exposure. This later CTSS-induced stimulation of PAR-2 protein synthesis may be required to replenish cellular PAR-2 after its initial cleavage and activation during acute CTSS treatment, thus restoring PAR-2 stores in the plasma membrane. In contrast TNF-α gene and protein expression gradually decreased after the initial peak at 2 h of CTSS treatment, suggesting the possibility of early induction of other proteins, which may inhibit TNF-α such as IL-6 [61].

Less extensive changes in protein expression in cell culture medium were detected in HCE-T cells treated with PAR-2 siRNA relative to scrambled siRNA and exposed to chronic CTSS for 24 h, relative to acute CTSS treatment. IL-6 and TNF-α release were reduced, continuing the trend at 4 h, while MMP-9 release was also reduced. Since the expression of these effectors is interrelated, it is unclear whether the more sustained effects on IL-6, TNF-α, and MMP-9 are truly due to PAR-2 or whether the effect at 24 h is related to accumulated changes from the acute burst. No changes in protein content in cell lysates of any CTSS-sensitive proteins were observed in 24 h CTSS-treated cells with PAR-2 siRNA relative to scrambled siRNA. However, CTSS-dependent upregulation of gene expression of all proteins of interest examined at 24 h was significantly reduced by siRNA to PAR-2, suggesting that it may play a role in the cycle of chronic inflammation, perhaps mediated by one of the acute phase pro-inflammatory cytokines.

The acute phase of IL-8 release induced by CTSS at 4 h was not reduced by the partial knockdown of PAR-2, suggesting that this effect is independent of PAR-2. One possible mechanism is through IL-36γ, a member of the IL-1 family [62] abundant in epithelia, neurons, glia, dendritic cells, and macrophages. IL-36γ is highly increased in blood and salivary glands of primary SS patients and correlated with IL-17A and IL-22 serum levels [63]. A recent study has shown that CTSS cleavage of IL-36γ induces secretion of IL-8 in human keratinocytes [54].

Recent studies have suggested the potential of PAR-2 and CTSS inhibitors as anti-inflammatory therapies. The CTSS inhibitor (RO5459072, IC_50_ 0.1 nM and 0.3 nM toward human and murine CTSS, respectively) is a reversible CTSS inhibitor due to high affinity formation of a covalent bond between the nitrile moiety of the inhibitor and the catalytic residue of CTSS. It reduces systemic autoimmunity and autoimmune nephritis in a murine model of systemic lupus erythematosus and SS [39]. Another study from the same group showed that the use of another CTSS inhibitor (RO5461111, IC_50_ 0.4 nM and 0.5 nM toward human and murine CTSS, respectively), a competitive reversible inhibitor [64], with and without the PAR-2 inhibitor, GB83 which inhibits trypsin cleavage of PAR-2, reduced markers of diabetic nephropathy and diabetic retinopathy in type 2 diabetic (db/db) mice [38]. In addition, the inhibition of CTSS by MV026031 (K_i_ 47 nM and 22 nM affinity toward human and murine CTSS, respectively) or antagonism of PAR-2 by GB88 (IC_50_ 1 µM), which inhibits trypsin-mediated PAR-2 cleavage and has shown potential for inhibition of CTSS-mediated PAR-2 cleavage, can suppress formalin-induced endogenous CTSS activity leading to hyperalgesia in mice [37]. Another study using GB88 showed that it was also able to inhibit PAR-2 activation of nociceptors by trypsin, elastase, and CTSS to abolish extracellular CTSS-induced edema and attenuate stimulated mechanical and thermal hyperalgesia in mice [65]. Therefore, blockage of CTSS or PAR-2 using CTSS inhibitors or CTSS-specific PAR-2 inhibitors, especially as more selective agents to CTSS-mediated PAR-2 cleavage become available in future, may have therapeutic potential for reducing ocular surface inflammation in SS patients.

In conclusion, we demonstrate that the highly increased CTSS characteristic of SS patient tears can induce pro-inflammatory cytokines including IL-8, IL-6, TNF-α, and IL-1β, as well as MMP-9 and CTSS itself. These inflammatory mediators may create a cycle of escalating inflammation on the ocular surface of SS patients. A possible mechanism behind CTSS induction of these inflammatory mediators, in the acute stage, may involve corneal and ocular surface PAR-2.

## 4. Materials and Methods

### 4.1. Reagents

Human recombinant CTSS and the Cathepsin S Activity Assay Kit were purchased from Biovision (Milpitas, CA, USA). The RNase^®^ plus mini kit, Hs_F2RL1_5 FlexiTube siRNA and AllStar Negative Control siRNA were from Qiagen (Germantown, MD, USA). The RT kit for reverse transcriptase, human IL-1β, IL-8, IL-6, TNF-α, MMP-9, CTSS, PAR-2 and GAPDH (endogenous control) primers, and master mix were purchased from Applied Biosystems (Grand Island, NY, USA). The CytoSelect^TM^ Cell viability and cytotoxicity assay kit was from Cell Biolabs, Inc. (San Diego, CA, USA). The human V-plex pro-inflammatory cytokine panel 1 kit was from Meso Scale Discovery (MSD^®^) (Rockville, MD, USA). The human IL-6 and IL-1β ELISA kits, lipofectamine RNAiMAX transfection reagent, Opti-Mem Reduced Serum Medium, the BLOCK-IT^TM^ Fluorescent Oligo labeled with FITC for transfection efficiency of siRNA, the mouse anti-GAPDH monoclonal antibody (Cat.#MA5-15738) used for Western Blotting, and Micro BCA reagents were from Thermo Fisher Scientific (Rockford, IL, USA). The human IL-8 ELISA kit was from Innovative Research (Novi, MI, USA). The human PAR-2 ELISA kit was purchased from Cloud-Clone Corp. (Katy, TX, USA) and the human Magnetic Luminex Assay Plex to measure protein expression of IL-1β, IL-8, IL-6, TNF-α, MMP-9, and CTSS was purchased from R&D Systems (Minneapolis, MN, USA). The rabbit anti-PAR-2 polyclonal antibody (Cat.#ab128628) used for immunofluorescence and the rabbit anti-PAR-2 monoclonal antibody (Cat.#ab180953) used for Western blotting were from Abcam (Cambridge, MA, USA). Alexa Fluor^®^ 488 donkey anti-rabbit secondary antibody (Cat.#A-21206), rhodamine-phalloidin (Cat.#A22287), and ProLong^®^ Gold Antifade Mounting Medium were from Invitrogen (Carlsbad, CA, USA). DAPI nucleic acid stain was from Molecular Probes, Inc. (Eugene, OR, USA). IRDye^®^ 800CW Goat anti-rabbit (Cat.#926-32211) and IRDye^®^ 680RD Donkey anti-mouse (Cat.#925-68072) were purchased from LICOR (Lincoln, NE, USA), and were used as secondary antibodies for Western Blotting. CHAPS was purchased from Sigma–Aldrich (St. Louis, MO, USA), while Tris-HCL, sodium chloride and acetone were from J.T. Baker (Center Valley, PA, USA). Bio-Rad protein assay dye reagent concentrate was from Bio-Rad (Hercules, CA, USA). Bovine serum albumin (BSA) was from Calbiochem^®^ (Billerica, MA, USA).

### 4.2. Calculation of Human Recombinant CTSS Dosage for Treatment of Human Corneal Epithelial Cells

Human recombinant CTSS at the same activity level detected in the 90th–95th percentile in tears of SS patients [59] and as used for other in vitro biochemical studies on SS tear properties [9] were used to treat a human corneal epithelial cell line (HCE T-cell). The 90th–95th percentile of enzymatic CTSS activity corresponded to 18,000 RFU (Relative Fluorescence Units) or 300 RFU per minute. Because the specific activity of the recombinant human enzyme varied by lot and also with storage time, a standard curve to determine the Relative Fluorescence Unit (RFU) and the concentration of recombinant human CTSS to achieve this activity was conducted for each experiment using the CTSS activity assay kit. Recombinant human CTSS was diluted into 5 concentrations: 2.5, 5.0, 12.5, 25, and 50 nM. 50 µL from each concentration was added into 2 wells of a 96-well plate. 50 µL of CTSS reaction buffer and 2 µL of substrate (Z-VVR-AFC) were added into each well. Then, 2 µL of CTSS inhibitor was added into one of the two wells as a negative control. The plate was incubated at 37 °C for 1 h and the fluorescence intensity read using a microplate spectrofluorometer (SpectraMax Gemini Plate Reader, Molecular Devices, Sunnyvale, CA, USA) with 400/505 nm excitation/emission filters. The concentration of human recombinant CTSS that showed enzymatic activity at 18,000 RFU or 300 RFU per minute was used to treat HCE T-cells in 500 µL of cell medium in a 12-well plate and in 700 µL of cell medium in a 6-well plate.

### 4.3. Cell Culture

A human corneal epithelial cell line, HCE-T cells, transformed with Simian virus 40-Adeno vector [66], was obtained from the RIKEN Cell Bank, Japan (Cat. # RCB2280). Medium for cell culture was prepared according to Cell Bank instructions and consisted of 250 mL of keratinocyte-SFM (KSFM) supplemented with 40 µL of human recombinant EGF (rEGF), 1 mL of Bovine Pituitary Extract (BPE), and 30 µL of gentamycin (Gibco^®^, Life Technologies, Grand Island, NY, USA). Cells were cultured to 80–90% confluence and split with 0.25% Trypsin-EDTA (Mediatech, Inc., Manassas, VA, USA). 10% of Heat-inactivated fetal bovine serum (Thermo Fisher Scientific, Rockford, IL, USA) in sterile PBS was used to quench trypsin digestion. All cells utilized were from passage 4–8.

### 4.4. siRNA Transfection

Cells were seeded in 6-well plates with KSFM with supplements but without gentamycin which can cause cell death during siRNA transfection. Cells at 50–60% confluency were transfected with PAR-2 siRNA or scrambled siRNA (a non-specific random sequence siRNA used as a negative control). 5 µL of Lipofectamine RNAiMAX reagent was diluted with 120 µL of Opti-Mem medium to make 125 µL of diluted Lipofectamine reagent, while 25 pmol of siRNA was diluted with Opti-Mem medium to make a total volume equal to 125 µL of diluted siRNA. These mixtures were mixed (ratio 1:1) and incubated at room temperature for 10 min. After that, the 250 µL of siRNA-lipid complex was added to the cells and incubated at 37 °C for 48 h. After 48 h, PAR-2 gene and protein expression were analyzed to analyze the extent of inhibition of PAR-2 expression in cells transfected with PAR-2 siRNA relative to cells transfected with scrambled siRNA as described below.

### 4.5. Transfection Efficiency

Transfection efficiency of siRNA was determined with the BLOCK-iT Fluorescent Oligo labeled with FITC as previously reported [67]. Cells were transfected with 25 pmol of BLOCK-iT fluorescent Oligo and 5 µL of Lipofectamine RNAiMAX reagent as described above. After 48 h, cells were washed with PBS and transfection efficiency was semi-quantitatively determined by confocal fluorescence microscopy using a ZEISS LSM 800 confocal microscope (Carl Zeiss, Thornwood, NY, USA). For quantitative measurements, cells were detached using Trypsin-EDTA (Mediatech, Inc., Manassas, VA, USA) and suspended in 700 µL of PBS. Flow cytometry using a BD LSRFortessa^TM^ X-20 (BD Biosciences, San Jose, CA, USA) was used to detect the FITC-positive HCE-T cells.

### 4.6. Treatment of Cells with Recombinant Human CTSS

HCE-T cells were seeded in KSFM medium supplemented with BPE, rEGF, and gentamycin as described in 12-well plates. Cells were starved for 16–18 h with KSFM medium without any additives. Human recombinant CTSS was added to cells at the dose corresponding to 18,000 RFU per 500 µL of cell medium as described above and incubated for 15 min, 1, 2, 4, 8, and 24 h. Cell viability after 24 h of CTSS treatment was measured using MTT colorimetric detection with reagents provided with the CytoSelect^TM^ Cell viability and cytotoxicity assay kit. For viability assays, cells were seeded in a 24-well plate and treated according to the manufacturer’s protocol. The plate was read using a SpectraMax iD3 (Molecular Devices, San Jose, CA, USA). For treatment of cells with heat-inactivated CTSS, active recombinant CTSS was heat-inactivated for 30 min at 90 °C and the loss of activity was confirmed using the CTSS activity assay kit. HCE T-cells were then treated with an equivalent starting activity of active versus heat-inactivated CTSS for 4 h. Gene expression of various pro-inflammatory cytokines and other markers were evaluated under each treatment condition as described below. For treatment of siRNA-transfected cells with CTSS, cells which were already transfected with siRNA was treated with human recombinant CTSS at the dose corresponding to 18,000 RFU per 700 µL of cell medium for 4 and 24 h. After 4 and 24 h of CTSS treatment, IL8, IL-6, TNF-α, and IL-1β, CTSS and MMP-9 gene and protein expression were measured as described below. 

### 4.7. Gene Expression

RNA was prepared from HCE-T cells using the RNeasy^®^ Plus Mini Kit. Reverse transcription reactions were performed with 1 µg of total RNA per 50 µL of reaction volume, which was composed of TaqMan^®^ Reverse Transcription Reagents to obtain cDNA from RNA using the GeneAmp^®^ PCR System 9700 (Applied Biosystems, Grand Island, NY, USA) with incubation at 25 °C for 10 min, then, at 48 °C for 30 min, and finally terminated by incubation at 95 °C for 5 min. Real-time quantitative polymerase chain reaction (qPCR) was conducted using an ABI 7900HT Fast Real-Time PCR System according to published procedures [68]. Briefly, samples were preheated at 95 °C for 10 min, followed by 40 cycles of 95 °C for 15 s and 60 °C for 1 min. GAPDH (Hs02786624_g1) was run as the internal control and IL-1β (Hs00174097_m1), IL-8 (Hs00174103_m1), IL-6 (Hs00985639_m1), TNF-α (Hs00174128_m1), MMP-9 (Hs00234579_m1), CTSS (Hs00175407_m1), and PAR-2 (Hs00608346_m1) primers were used to measure gene expression. The reaction conditions and calculation methods were as described previously [68].

### 4.8. Multiplex Assay and ELISA Methods for Measurement of Pro-Inflammatory Cytokines in HCE-T Cell Medium and Lysates

The cell medium was collected and then centrifuged at 500× *g* for 10 min (Z 216 MK-2 High Capacity Refrigerated Microcentrifuge, Edison, NJ, USA). Supernatants were kept on ice and concentrated by centrifugal filters (regenerated cellulose 10,000 NMWL, Burlington, MA, USA). The total protein concentration in the culture medium was measured using the Bio-Rad assay. For measurement of protein content in lysates, cells were lysed with lysis buffer (150 mM NaCl, 20 nM Tris base pH 7.5, 1 mM EDTA, 1 mM EGTA, 1% Triton X-100) [68] containing protease inhibitor cocktail as previously described [58] and were also incubated on ice for 30 min with vortexing every 10 min. Samples were centrifuged at 14,000× *g* for 15 min at 4 °C. Supernatants were collected and concentrated using centrifugal filters. The total protein concentration in cell lysates was measured using the BCA assay. The manufacturer’s protocol for the MSD^®^ MULTI-SPOT Assay System (Rockville, MD, USA) was followed to analyze the expression of 10 pro-inflammatory cytokines. The multiplex plate was read with an MSD^®^ Sector Imager 2400A Imaging System (Rockville, MD, USA). A standard curve for each cytokine was created. IL-6 and IL-8 protein expression in cell medium were measured separately using human ELISA kits according to the protocols provided and read using a SpectraMax iD3 (Molecular Devices, San Jose, CA, USA).

### 4.9. ELISA Method for Measurement of CTSS in HCE-T Cell Lysates

Cells were washed twice with PBS, then scraped gently into PBS for centrifugation at 1000× *g* for 5 min. The supernatant was removed and the cell pellets were resuspended with lysis buffer (without protease inhibitor cocktail). Then, cell lysates were prepared using the same method as for measurement of pro-inflammatory cytokines. The human CTSS ELISA kit with the protocol provided were used and the plate was read using a SpectraMax iD3 (Molecular Devices, San Jose, CA, USA). The total protein concentration in cell lysates was measured using the BCA assay.

### 4.10. ELISA Method for Measurement PAR-2 in HCE-T Cell Lysates

Cell lysates were prepared by lysing cells with lysis buffer containing protease inhibitor cocktail as previously described [58] and were also incubated on ice for 30 min with vortexing every 10 min. Samples were centrifugated at 1500× *g* for 10 min at 4 °C. Supernatants were collected and concentrated using centrifugal filters. The human PAR-2 ELISA kit and the protocol provided were used and the plate was read using a SpectraMax iD3 (Molecular Devices, San Jose, CA, USA). The total protein concentration in cell lysates was measured using the BCA assay.

### 4.11. Multiplex Assay and ELISA Methods for Measurement of Pro-Inflammatory Cytokines, CTSS, and MMP-9 in siRNA Transfected HCE-T Cells

For cells treated with siRNA and then exposed without and with CTSS, total CTSS, MMP-9, IL-6, IL-8, IL-1β, and TNF-α were quantified in both media and cell lysates using a Human Magnetic Luminex Assay, a bead-based multiplex ELISA kit (LXSAHM; R&D systems, Minneapolis, MN, USA) according to the manufacture’s protocol. Data from the multiplex ELISA was acquired on a validated and calibrated Bio-Plex Suspension Array 200 system (MAPTM Technology; Austin, TX, USA) and analyzed using Bio-Plex Manager 6.1 software (Austin, TX, USA). IL-6 and IL-1β protein expression in cell medium from cells treated with siRNA and exposed without and with CTSS were measured separately using human ELISA kits according to the protocol provided and read using SpectraMax iD3 (Molecular Devices, San Jose, CA, USA), since their range exceeded that of the other components measured by multiplex ELISA. The protein value acquired for each analyte was normalized to total protein concentration.

### 4.12. Immunofluorescence of PAR-2

HCE T- cells were seeded on coverslips in 12-well plates. Then, cells were washed with PBS and incubated with acetone at −20 °C for 10 min. After that, cells were washed with PBS and gently shaken with 1% BSA for 1 h at room temperature. Cells were then incubated with rabbit anti-PAR-2 polyclonal antibody at 1:50 dilution in BSA for 1 h at 37 °C. After that, cells were washed with PBS for 5 min, 3 times. Rhodamine-phalloidin was then added at 1:200 dilution, DAPI at 1:2000 dilution, along with Alexa Fluor^®^ 488 donkey anti-rabbit secondary antibody at 1:100 dilution and incubated for 1 h at 37 °C. Samples were mounted using ProLong^®^ Gold Antifade (Carlsbad, CA, USA) mounting medium and imaged by confocal fluorescence microscopy using a ZEISS LSM 800 confocal microscope (Carl Zeiss, Thornwood, NY, USA).

### 4.13. CTSS Activity in HCE-T Cell Lysates

Cell medium was removed and cells were washed twice with PBS, then scraped gently into PBS for centrifugation at 1000× *g* for 5 min. The supernatant was removed and cell lysates were prepared in the cell lysis buffer provided in the CTSS assay kit and incubated on ice for 30 min. Cell lysates were vortexed every 10 min and then, centrifuged at 14,000× *g* for 15 min at 4 °C Supernatants were concentrated using centrifugal filters. CTSS activity in cell lysates was determined with the CTSS activity assay kits described above, according to the manufacturer’s instructions, and the enzymatic reaction was incubated at 37 °C for 1 h. The quantity of the resulting fluorescent products was measured in a microplate spectrofluorometer (SpectraMax Gemini Plate Reader, Molecular Devices, Sunnyvale, CA, USA) with 400/505 nm excitation/emission filters. Activity of CTSS was measured as relative fluorescence units. Total protein concentration in cell lysates was measured using the BCA protein assay and plates were read using a Tecan GENios Plus Microplate Reader (Meilen, Zurich, Switzerland) with absorbance set to 595 nm. The unit of total protein concentration was µg/µL.

### 4.14. Western Blotting for PAR-2

Cells were removed by scraping in CHAPS lysis solution (30 mM CHAPS in TBS/20 mM Tris HCl, 150 mM NaCl, pH 8.0). Cells were further lysed by passing through a 23-gauge needle 10 to 15 times. Cell lysates were left at 4 °C for 1 h. Post-incubation, insoluble debris was removed from the lysate by centrifugation at 15,000× *g* at 4 °C for 5 min. The supernatants were collected and concentrated using centrifugal filters. The protein concentration of the concentrated lysates was measured using the BCA assay. Lysate samples were left in a reducing dye containing β-mercaptoethanol for 1 h at room temperature. Precast 10% PAGEr™ EX Gels (Lonza, Rockland, ME, USA) ware used for resolution of samples. 40 µg of lysate/β-mercaptoethanol/dye mix were loaded into each well, and run at 80 V, 4 °C for 2 h. Proteins were then transferred to nitrocellulose membranes with the iBlot 2 dry blotting system (ThermoFisher Scientific, Rockford, IL, USA). Membranes were incubated at room temperature in fluorescent blocking buffer (Rockland Immunochemicals Inc, Limerick, PA, USA). After blocking, the membranes were gently shaken overnight in blocking buffer containing primary PAR-2 antibody at a 1:750 dilution (and/or 1:1000 dilution for GAPDH) at 4 °C. On the second day, the membranes were washed 3 times, each 5 min, with 0.2% Tween containing Tris-buffered saline. The membranes were then incubated in secondary antibody containing blocking buffer (1:2000 dilution) at room temperature, for 1 h. After washing with 0.2% Tween containing Tris-buffered saline again as above, the membranes were imaged with an Odyssey Licor imaging system (LI-COR Biotechnology, Lincoln, NE, USA).

### 4.15. Statistics

All statistical analyses were performed using Graphpad Prism software (Graphpad, San Diego, CA, USA). D’Agostina-Pearson, Shapiro-Wilk, and Kolmogorov-Smirnov were used to define normality of data. For gene and protein expression experiments that were analyzed at several time points, one-way ANOVA with Dunnett’s multiple comparison was used to compare each CTSS treatment group with a single control group [69]. For measurement of IL-6 and IL-8 gene expression in untreated, heat-inactivated CTSS and active CTSS-treated cells, one-way ANOVA with Tukey’s multiple comparison was used to compare the effect between three groups. For gene, protein expression, and enzymatic activity experiments that were analyzed and compared between an experimental group and a single control group, a two-tailed, unpaired Student’s t-test was used to compare between the experimental group and the control group.

## Figures and Tables

**Table 1:** Decreased pro-inflammatory cytokine and protease gene expression in human corneal epithelial cells transfected with PAR-2 siRNA after 4 h and 24 h of CTSS treatment.

Gene Expression	Relative Expression					
	4 h of CTSS Treatment			24 h of CTSS Treatment		
	Scrambled siRNA	PAR-2 siRNA	*p* Value	Scrambled siRNA	PAR-2 siRNA	*p* Value
***IL-8***	1.007 ± 0.084	1.109 ± 0.130	ns	**1.029 ± 0.180**	**0.348 ± 0.057**	*
***IL-6***	**1.007 ± 0.085**	**0.525 ± 0.079**	*	**1.000 ± 0.021**	**0.090 ± 0.007**	****
***IL-1β***	1.187 ± 0.493	0.546 ± 0.153	ns	**1.032 ± 0.183**	**0.222 ± 0.068**	*
***TNF-α***	**1.048 ± 0.224**	**0.382 ± 0.083**	*	**1.007 ± 0.087**	**0.267 ± 0.040**	**
***CTSS***	1.012 ± 0.115	1.156 ± 0.263	ns	**1.017 ± 0.131**	**0.289 ± 0.059**	**
***MMP-9***	1.050 ± 0.243	0.723 ± 0.112	ns	**1.011 ± 0.104**	**0.099 ± 0.034**	**

The amount of CTSS added corresponded to an activity level found in the 90th–95th percentile of SS patients (18,000 RFU, added to 700 µL of cell medium), as described in detail in **Methods**. *n* = 3 samples/group, * *p* ≤ 0.05, ** *p* ≤ 0.01, **** *p* ≤ 0.0001, ns = not significant, data are represented as mean ± SEM, and a two-tailed, unpaired Student’s *t*-test was used to compare PAR-2 siRNA transfected to scrambled siRNA transfected cells.

**Table 2:** Pro-inflammatory cytokine and protease protein expression in cell culture medium and cell lysates of human corneal epithelial cells transfected with PAR-2 siRNA after 4 h and 24 h of CTSS treatment.

Protein Expression	Protein Expression in Cell Lysates (pg/mg protein)					
	4 h of CTSS Treatment			24 h of CTSS Treatment		
	Scrambled siRNA	PAR-2 siRNA	*p* Value	Scrambled siRNA	PAR-2 siRNA	*p* Value
***Cell Culture Medium***						
**IL-8**	18759 ± 61.6	19800 ± 472.2	ns	15254 ± 445	20377 ± 289.2	ns
**IL-6 ^a^**	**79003 ± 12649**	**26608 ± 9138**	*	**42730 ± 2075**	**17810 ± 844.4**	***
**IL-1β ^a^**	**1.240 ± 0.1052**	**0.738 ± 0.095**	*	1.826 ± 0.235	1.302 ± 0.072	ns
**TNF-α**	**105.8 ± 7.221**	**43.52 ± 1.785**	**	**44.64 ± 2.173**	**22.09 ± 0.662**	***
**MMP-9**	**10834 ± 881**	**5613 ± 48.90**	**	**34666 ± 836**	**19702 ± 3355**	*
***Cell Lysates***						
**IL-8**	147.5 ± 16.61	115.7 ± 10.92	ns	108.8 ± 18.13	113.8 ± 21.64	ns
**IL-6**	**8.784 ± 0.560**	**3.862 ± 1.133**	*	3.155 ± 0.746	1.548 ± 0.231	ns
**IL-1β**	138.0 ± 7.153	94.75 ± 15.88	ns	62.99 ± 11.64	36.33 ± 7.632	ns
**TNF-α**	0.836 ± 0.056	0.778 ± 0.145	ns	0.794 ± 0.039	0.669 ± 0.117	ns
**CTSS**	25.16 ± 5.543	33.53 ± 6.185	ns	35.60 ± 6.494	34.30 ± 8.386	ns
**MMP-9**	**6160 ± 329.0**	**3322 ± 309.5**	**	10628 ± 1137	5980 ± 1390	ns

The amount of CTSS added corresponded to an activity level found in the 90th–95th percentile of SS patients (18,000 RFU, added to 700 µL of cell medium), as described in detail in **Methods**. *n* = 3 samples/group, * *p* ≤ 0.05, ** *p* ≤ 0.01, *** *p* ≤ 0.001, ns = not significant, ^a^ = individual ELISA kit, data are represented as mean ± SEM, and a two-tailed, unpaired Student’s *t*-test was used to compare PAR-2 siRNA transfected to scrambled siRNA transfected cells. We did not measure CTSS protein expression in cell culture medium since we could not distinguish added recombinant CTSS from that released from the cells.

## Abbreviations

SS	Sjögren’s syndrome
LG	Lacrimal gland
CTSS	Cathepsin S
IL	Interleukin
TNF	Tumor necrosis factor
TGF	Transforming growth factor
IFN	Interferon
MMP	Matrix metalloproteinase
NOD	Non-obese diabetic
PAR	Protease-activated receptor
GPCR	G-protein-coupled receptor
HCE-T	Human corneal epithelial cell line
SEM	Standard error of the mean
Th	T helper cell
siRNA	Small interfering RNA
CD	Cluster of differentiation
